# Diffusion-weighted whole-body magnetic resonance imaging with background body signal suppression was useful in a patient with isolated myocardial abscess confined to the right atrial wall: a case report

**DOI:** 10.1186/s12872-023-03366-w

**Published:** 2023-07-05

**Authors:** Marohito Nakata, Naoko Yokota, Tsuneaki Kenzaka

**Affiliations:** 1 Department of Cardiology, Urasoe General Hospital, Urasoe, Japan; 2 Department of Cardiology, Naha City Hospital, Naha, Japan; 3 Division of Community Medicine and Career Development, Kobe University Graduate School of Medicine, Hyogo, Japan; 4 Division of Community Medicine and Career Development, Kobe University Graduate School of Medicine, 2-1-5, Arata-cho, Hyogo-ku, Kobe, 652-0032 Hyogo Japan

**Keywords:** Myocardial abscess, Infective endocarditis, Diffusion-weighted whole-body magnetic resonance imaging, Background body signal suppression

## Abstract

**Background:**

Myocardial abscess is often associated with infective endocarditis (IE), and isolated myocardial abscess without IE is rare. Echocardiography and computed tomography (CT) are often used to diagnose myocardial abscess; however, to the best of our knowledge, diffusion-weighted whole-body magnetic resonance imaging with background body signal suppression (DWIBS) has not been used. Here, we present a case of myocardial abscess without IE that was diagnosed using DWIBS.

**Case presentation:**

: A 72-year-old Japanese man with a history of hypertension, dyslipidemia, and retinitis pigmentosa presented to our hospital with malaise and a fever lasting 10 days. Blood test results showed elevated inflammatory marker levels (white blood cell count 18,700/µL and C-reactive protein level 23.0 mg/dL). Infection was suspected; however, the source of the infection could not be identified. DWIBS, which was performed on day 7 of admission to determine the source of infection, showed a high signal surrounding the right wall, suggesting inflammation. Contrast-enhanced CT performed on day 1 of hospitalization revealed a low-density area in the same region; however, the pathological implications of this finding could not be determined. Based on DWIBS findings, we concluded that the condition presented as a myocardial abscess that was confined specifically to the right atrial wall. Three sets of blood cultures revealed negative findings, and echocardiography showed no vegetation or valve regurgitation. Therefore, the patient was diagnosed with an isolated myocardial abscess uncomplicated with IE. An electrocardiogram on admission showed no P waves, and the patient had a junctional rhythm. However, on day 20 of hospitalization, he developed a complete atrioventricular block. After complete myocardial abscess healing following antibiotic treatment was confirmed, the patient underwent pacemaker implantation. Ten months after surgery, the patient had no signs of infection recurrence.

**Conclusions:**

Based on history and physical examination alone, diagnosis of an isolated myocardial abscess can be challenging. In addition to CT and echocardiography, DWIBS might be helpful for the diagnosis of myocardial abscesses.

## Background

Myocardial abscesses are usually associated with infective endocarditis (IE), with the heart valve ring areas being their primary predilection sites [1]. Due to their rare occurrence, isolated myocardial abscesses without IE are usually not described in the guidelines for the prevention and treatment of IE published by the Japanese Society of Cardiology [1, 2], limiting their diagnosis and treatment. An isolated myocardial abscess is not associated with the valvular annulus and occurs when local bacterial myocarditis is caused by bloodstream infection and progresses to liquefaction and necrosis [3]. In the past, myocardial abscesses were primarily discovered during autopsies; however, at present, noninvasive imaging modalities such as echocardiography, computed tomography (CT), and magnetic resonance imaging (MRI) can be used for diagnosis. However, despite advances in diagnostic methods, identification of myocardial abscesses is challenging because of the low sensitivity of current diagnostic methods. Further, to the best of our knowledge, diffusion-weighted whole-body MRI with background body signal suppression (DWIBS) has not been used for the diagnosis of myocardial abscesses. Here, we report a case of myocardial abscess without IE that was diagnosed using DWIBS.

### Case presentation

A 72-year-old Japanese man presented to our hospital with a fever and malaise. He had a history of hypertension, dyslipidemia, and retinitis pigmentosa. Ten days before visiting our hospital, he visited his family doctor for a fever. Since he showed negative findings for severe acute respiratory syndrome coronavirus 2 on the polymerase chain reaction test, he was prescribed antipyretics (acetaminophen 500 mg per dose). However, the fever persisted, and the malaise did not improve; therefore, he revisited his family doctor. He was referred to our hospital because his blood test showed elevated inflammatory marker levels (white blood cell count 18,700/µL and C-reactive protein level 23.0 mg/dL). The patient was admitted to our hospital because the source of the fever could not be identified based on medical history, physical examination findings, and blood test results (Table 1).

**Table 1:** Laboratory data at the first visit

Parameter	Recorded value	Standard value
White blood cell count	19,600/µL	3300–8600/µL
Hemoglobin	11.9 g/dL	11.5–15.0 g/dL
Platelet count	54.6 × 10^4^/µL	15–35 × 10^4^/µL
C-reactive protein	27.67 mg/dL	≤ 0.14 mg/dL
Total protein	6.8 g/dL	6.6–8.1 g/dL
Albumin	2.6 g/dL	4.1–5.1 g/dL
Total bilirubin	0.7 mg/dL	0.4–1.5 mg/dL
Aspartate aminotransferase	142 U/L	13–30 U/L
Alanine aminotransferase	101 U/L	7–23 U/L
Lactase dehydrogenase	245 U/L	124–222 U/L
γ-Glutamyl transpeptidase	263 U/L	13–64 U/L
Blood urea nitrogen	25.4 mg/dL	8–20 mg/dL
Creatinine	1.20 mg/dL	0.46–0.79 mg/dL
Sodium	137 mEq/L	138–145 mEq/L
Potassium	5.4 mEq/L	3.6–4.8 mEq/L
Chloride	101 mEq/L	101–108 mEq/L
Glucose	105 mg/dL	73–109 mg/dL
Thyroid-stimulating hormone	1.504 µIU/L	0.34–4.22 µIU/L
Free T4	0.94 ng/dL	0.77–1.74 ng/dL
Total cholesterol	94 mg/dL	142–248 mg/dL
Triglyceride	58 mg/dL	30–117 mg/dL
High-density lipoprotein cholesterol	23 mg/dL	48–103 mg/dL
Low-density lipoprotein cholesterol	59 mg/dL	65–163 mg/dL
Hemoglobin A1c	5.9%	4.9–6.0%

### Investigation

**Fig. 1:**
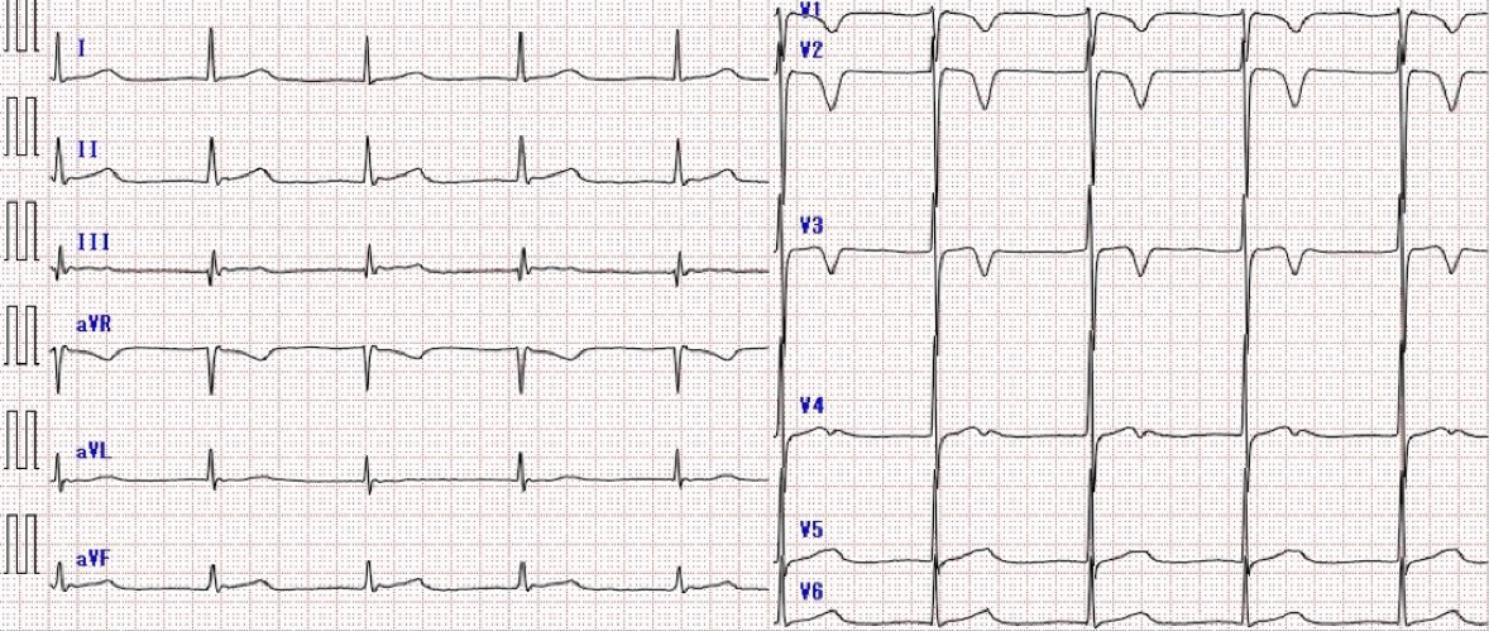
Electrocardiogram on admission No P wave is detected; therefore, a diagnosis of junctional rhythm is made

**Fig. 2:**
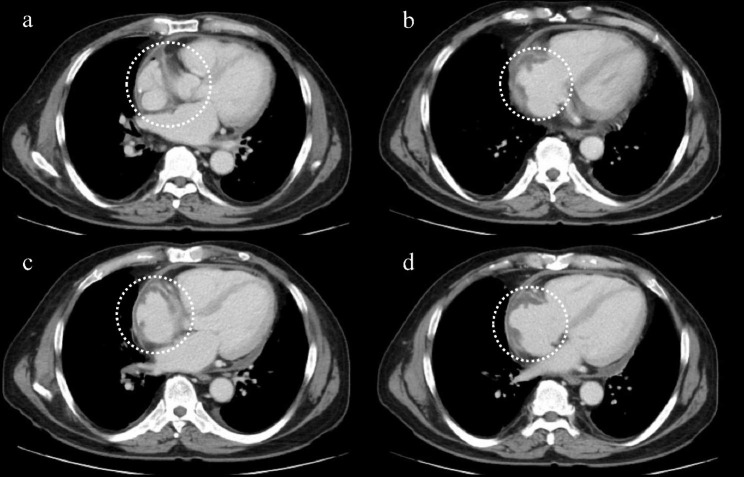
Contrast-enhanced computed tomography on admission The images are horizontal sections from **a**, **b**, **c**, and **d**, arranged from cranial to caudal order. Low-density area along the right atrial wall

The vital signs at the time of examination were as follows: blood pressure, 90/51 mmHg; heart rate, 66 beats/min; respiratory rate, 23 breaths/min; body temperature, 36.3 °C; and partial pressure of oxygen, 98% (ambient air). Physical examination revealed good oral hygiene and no heart murmurs. Electrocardiography showed a junctional rhythm (Fig. 1). Contrast-enhanced CT revealed a low-density area along the wall of the right atrium (Fig. 2); however, it was difficult to determine the presence of inflammation.

### Differential diagnosis

**Fig. 3:**
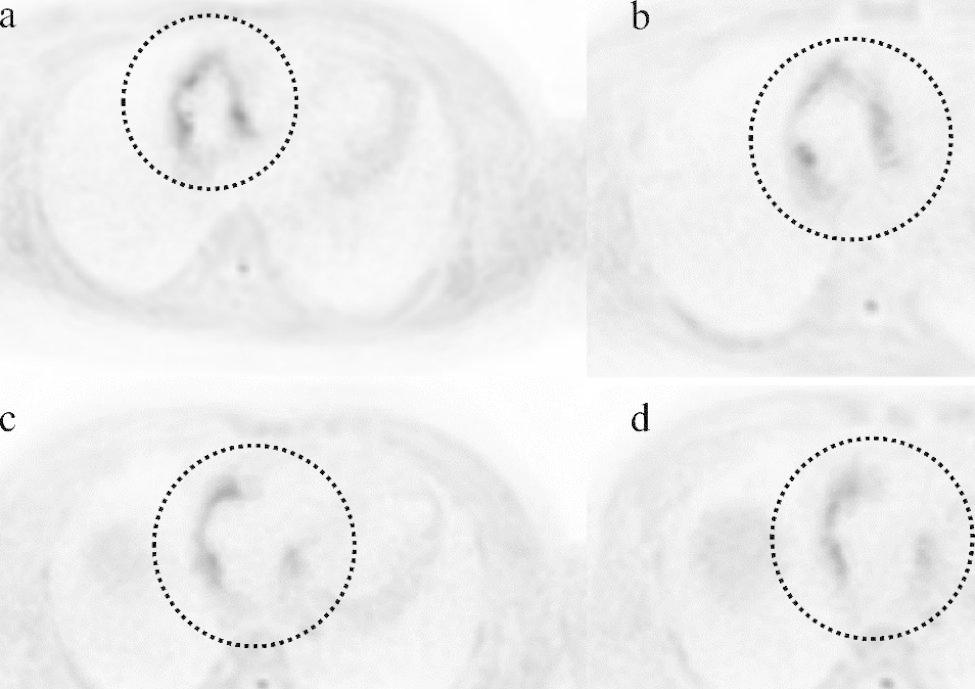
Diffusion-weighted whole-body magnetic resonance imaging with background body signal suppression The images are horizontal sections from a, b, c, and d arranged from cranial to caudal order. We could detect high-signal area, which CT was low density CT: computed tomography

A bacterial infection was suspected; however, the patient had no subjective symptoms, and the source of infection could not be identified on imaging. DWIBS from the chest to the abdomen was performed on day 7 of hospitalization, and a high signal was detected in the right atrial wall, which was consistent with findings of the low-density area on CT, suggesting inflammation (Fig. 3). DWIBS was performed using a 1.5 Tesla imager (Ingenia Ambition, Philips, Amsterdam, Netherlands), with the following sequence parameters: flip angle 90°, b value 1000 s/mm^2^, repetition time 5000–6000 msec, echo time of 120 msec, matrix size 112 × 256, field of view 460 × 460 mm, and section thickness 5 mm. Acquisitions were conducted during unimpeded respiration, with an average acquisition time of 166 s/section, encompassing the thoracic and abdominal regions. It is important to note that this imaging method is not specific to the heart. Blood cultures were performed on days 1, 6, and 9 of hospitalization, all yielding negative results. Echocardiography revealed no vegetation or valve regurgitation, and the patient did not meet the diagnostic criteria for IE [4]. Based on the CT and DWIBS results, the patient was diagnosed with myocardial abscess that was confined to the right atrial wall.

### Outcome and follow-up

**Fig. 4:**
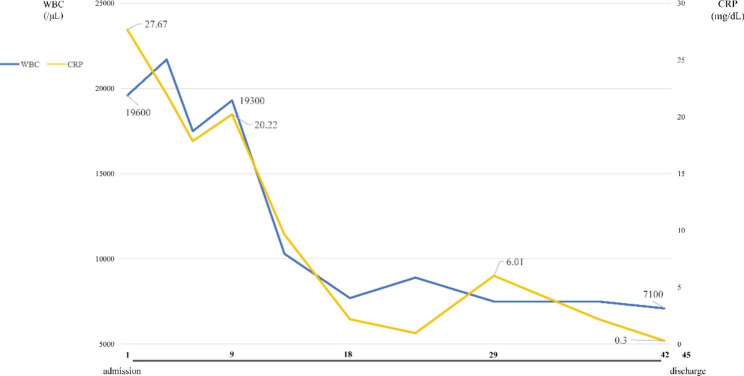
Trend in white blood cell count and C-reactive protein level Improvements are noted in both white blood cell count and C-reactive protein level after antibiotic treatment

**Fig. 5:**
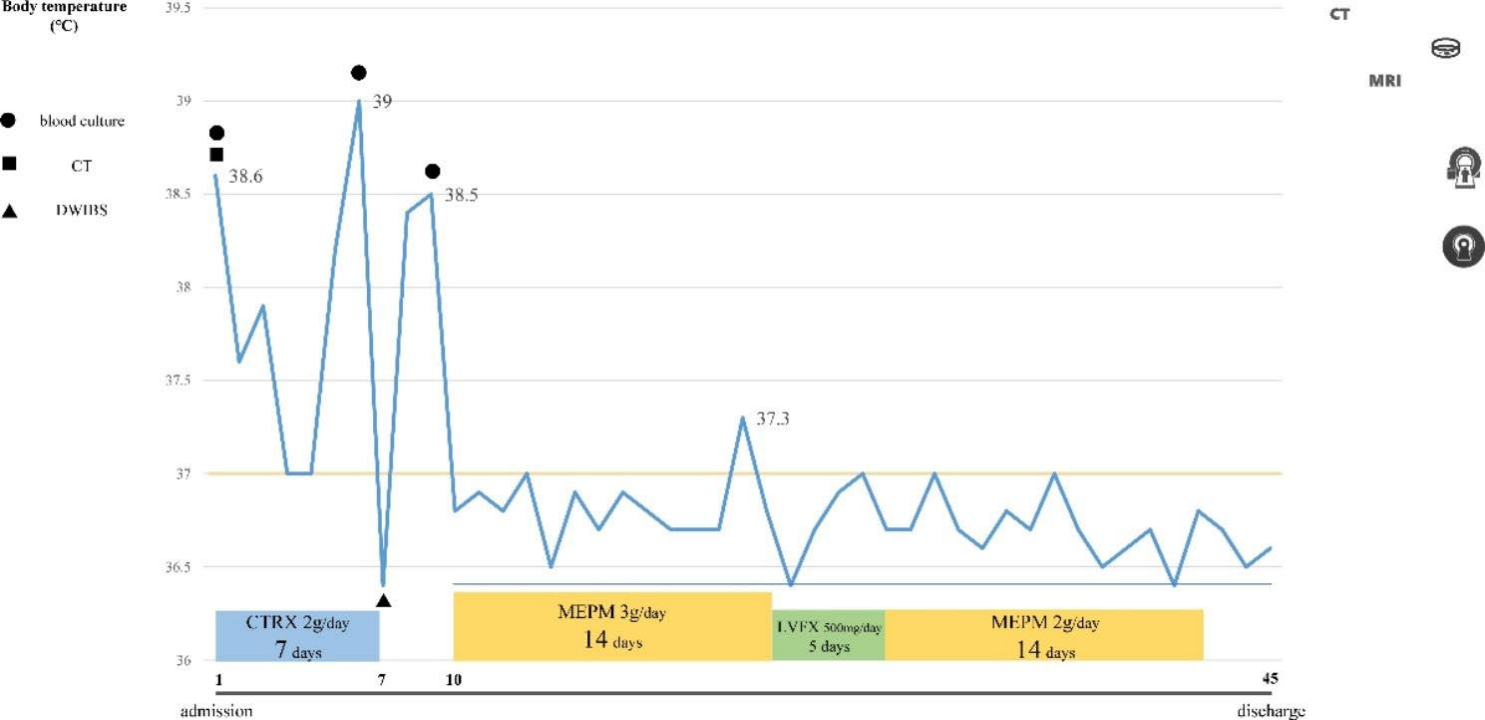
Clinical course showing the body temperature, type and dosage of antibiotic, and duration of administration The tests performed are also shown ●Blood cultures on days 1, 6, and 9 of hospitalization ■ Contrast-enhanced CT ▲DWIBS CTRX: ceftriaxone; MEPM: meropenem; LVFX: levofloxacin; DWIBS: diffusion-weighted whole-body magnetic resonance imaging with background body signal suppression; CT: computed tomography

Ceftriaxone sodium (1 g every 12 h, third-generation cephalosporin) was administered since admission; however, elevated white blood cell count and C-reactive protein level and fever did not resolve (Fig. 4). On day 7 of admission, DWIBS results led to a diagnosis of myocardial abscess; however, blood cultures were negative, and the organism causing the abscess was unknown. Since the fever persisted and blood test results did not improve, the patient was switched to meropenem (1 g every 8 h, carbapenem), which improved his condition. After 14 days of meropenem administration, the patient was switched to levofloxacin (500 mg every 24 h, new quinolone); however, because his C-reactive protein level increased again, he was switched back to meropenem (1 g every 12 h) for a total of 28 days (Figs. 4 and 5). Contrast-enhanced CT performed before discharge from the hospital confirmed shrinkage of the low-density area. An electrocardiogram on admission showed no P waves, and a junctional rhythm was noted; however, on day 20 of hospitalization, he developed a complete atrioventricular block. As there were no subjective symptoms associated with bradycardia, the patient was discharged from the hospital on day 42 after admission; he was refrained from pacemaker implantation, as the conduction disturbance was thought to be caused by a myocardial abscess. However, 1 month after discharge, he visited our hospital complaining of dizziness and lightheadedness.

Contrast-enhanced CT showed that the low-density area had disappeared entirely, and the myocardial abscess had healed. After confirming the healing of the myocardial abscess, we implanted a pacemaker. Ten months after surgery, the patient had no signs of infection recurrence.

## Discussion and conclusions

### First and second novelty

This study reports a case of an isolated myocardial abscess that was localized exclusively within the right atrial wall. Despite its rarity as a cause of fever [1], isolated myocardial abscess should be considered seriously. To the best of our knowledge, this is the first report of myocardial abscess diagnosed using DWIBS.

### Significance of first and second novelty

Due to its rare occurrence, isolated myocardial abscess without IE is rarely described in the guidelines for prevention and treatment of IE published by the Japanese Society of Cardiology [2], limiting its diagnosis and treatment. Decubitus ulcers, infections associated with burns, bronchiectasis, and sepsis due to thrombophlebitis in immunocompromised patients are associated with isolated myocardial abscesses [5]. There are reports of abscess formation at the site of myocardial infarction [6]; however, the present case occurred without any risk of abscess formation in the present case. Unlike abscesses associated with IE, isolated myocardial abscesses are not associated with the valve annulus [1] and are considered to be caused by local bacterial bloodstream infection, progressing to liquefaction necrosis [3]. In other words, sepsis triggers myocardial abscess formation; however, in the present case, three sets of blood cultures (two per time, six in total) revealed negative findings. Moreover, 47.1% of patients with culture-negative severe sepsis have a 1.75-fold higher odds ratio for death than those with culture-positive sepsis [7], and positive blood culture is not essential for the diagnosis of sepsis. The high mortality rate in blood culture-negative sepsis can be attributed to the challenges in selecting appropriate antibiotics and the delay or shortened duration of antibiotic therapy. We believe that our patient had culture-negative sepsis; therefore, we continued administering meropenem for 28 days. As a result, the fever subsided, and the subsequent resolution of the abscess was confirmed by contrast-enhanced CT. Surgical intervention might have been necessary if the fever or abscess persisted.

In the past, most myocardial abscesses were detected during an autopsy, but currently, noninvasive imaging techniques such as echocardiography, CT, and MRI can be used [1]. However, despite advances in diagnostic methods, identification of myocardial abscesses remains challenging. Echocardiography is useful for detecting myocardial abscesses, but the sensitivity of transthoracic echocardiography (TTE) is 50% and that of transesophageal echocardiography (TEE) is 90% [1]. TEE can detect valve annular abscess, valve perforation, fistula, and tendon rupture; however, evaluation of the right atrial wall is challenging due to its anatomical complexity. In the present case, we conducted TTE but found no abnormalities in the right atrium. TEE was not conducted in this case, as the use of DWIBS proved instrumental in the diagnostic process. DWIBS is based on diffusion-weighted imaging, which helps in visualizing the random motion of water molecules (Brownian motion) to enable diagnostic imaging [8]. DWIBS uses multiple signal averaging, fat suppression, and heavy diffusion weighting to acquire images without the respiratory restriction of the patient [8]. DWIBS provides a strong contrast between cancer and normal surrounding tissues, making it useful for cancer detection and staging. It has a high sensitivity, which is comparable to that of fluorodeoxyglucose positron emission tomography [9]. In addition, it can detect decreased diffusion motion associated with interstitial edema and necrosis as a signal enhancement. DWIBS can detect tumors and acute inflammation due to suppressed diffusion of water molecules [10]. Additionally, diffusion-weighted imaging can identify myocardial inflammation associated with acute myocarditis [11]. In the present case, DWIBS showed a high signal coinciding with the low-density area of contrast-enhanced CT; however, since the low-density area of CT disappeared afterward, the high signal of DWIBS was considered to indicate acute inflammation rather than a tumor. To date, myocardial abscess has been diagnosed using conventional T1- and T2-weighted imaging in only few cases [12–18]. However, to the best of our knowledge, no study has reported on the diagnosis of isolated myocardial abscesses using DWIBS.

### Reference to clinical utility

Morphological evaluation of myocardial abscesses, including location, size, and relationship to the coronary arteries, is essential for determining the course of treatment [1]. Treatment depends on the clinical course, imaging findings, and presence or absence of complications and varies from medical therapy to surgical drainage of the abscess and repair of the defect. In most cases, the abscess is small and improves with conservative treatment; however, complications such as fistula formation and myocardial rupture require caution [1]. DWIBS is effective not only in the diagnosis of a myocardial abscess but also in morphological evaluation. DWIBS is noninvasive, avoids radiation exposure, and allows imaging even in patients with poor renal function. In addition, the high signal detected by DWIBS decreases as inflammation improves, which is considered an indicator of inflammatory activity [10, 19]. Given the lack of reported cases regarding the use of DWIBS for diagnosing isolated myocardial abscesses, it is imperative to explore the sensitivity and specificity of this diagnostic modality in future studies. Previous research has reported that the sensitivity of abdominal ultrasound and DWIBS in diagnosing acute cholecystitis to range from 37.5 to 91%, and 90.9% respectively [19]. In the present case, contrast-enhanced CT was used to monitor the abscess due to normal renal function and lower imaging frequency. However, if renal function is impaired and frequent CT examination is required, DWIBS evaluation may be preferable as it poses less burden on patients.

## Conclusion

Despite its rarity, it is important to consider isolated myocardial abscess, it is important to consider isolated myocardial abscess as a cause of fever. In the absence of IE, DWIBS might be helpful in the diagnosis of isolated myocardial abscesses. This is the first report of a myocardial abscess diagnosed using DWIBS, highlighting the need for further investigation into its diagnostic performance.

## Data Availability

All data generated or analyzed during this study are included in this published article.

## Abbreviations

DWIBS: Diffusion-weighted whole-body magnetic resonance imaging with background body signal suppression
TTE: Transthoracic echocardiography
IE: infective endocarditis, CT:Computed tomography
MRI: Magnetic resonance imaging
TEE: Transesophageal echocardiography
